# Are Bone and Muscle Changes from POWER PE, an 8-month In-school Jumping Intervention, Maintained at Three Years?

**DOI:** 10.1371/journal.pone.0039133

**Published:** 2012-06-13

**Authors:** Benjamin K. Weeks, Belinda R. Beck

**Affiliations:** Centre for Musculoskeletal Research, Griffith University, Gold Coast, Queensland, Australia; INSERM U1059/LBTO, Université Jean Monnet, France

## Abstract

Our aim was to determine if the musculoskeletal benefits of a twice-weekly, school-based, jumping regime in healthy adolescent boys and girls were maintained three years later. Subjects of the original POWER PE trial (n = 99) were contacted and asked to undergo retesting three years after cessation of the intervention. All original measures were completed including: sitting height, standing height, weight, calcaneal broadband ultrasound attenuation (BUA), whole body, hip and spine bone mineral content (BMC), lean tissue mass, and fat mass. Physical activity was recorded with the bone-specific physical activity questionnaire (BPAQ) and calcium intake was estimated with a calcium-focussed food questionnaire. Maturity was determined by Tanner staging and estimation of the age of peak height velocity (PHV). Twenty-nine adolescents aged 17.3±0.4 years agreed to participate. Three years after the intervention, there were no differences in subject characteristics between control and intervention groups (p>0.05). Three-year change in weight, lean mass, and fat mass were similar between groups (p>0.05). There were no significant group differences in three-year change in BUA or BMC at any site (p>0.05), although the between-group difference in femoral neck BMC at follow-up exceeded the least significant change. While significant group differences were not observed three years after cessation of the intervention, changes in bone parameters occurred in parallel for intervention and control groups such that the original benefits of the intervention observed within the treatment group were sustained.

## Introduction

It is well recognised that efforts to prevent osteoporosis are likely to be more successful and economical than attempting to manage and rehabilitate fragility fractures. Childhood and adolescence are recognised as especially opportune times to intervene in order to maximise peak bone mass and strength during growth with the view to offset age-related loss. Many pediatric exercise interventions have elicited significant musculoskeletal benefits [1], [2], [3], [4], [5], [6], [7]. Whether those benefits are sustained in the longer term has yet to be confirmed.

The *Preventing Osteoporosis With Exercise Regimes in Physical Education* (POWER PE) trial was an 8-month in-school jumping intervention for adolescent boys and girls (mean age 13.8 years). Participants in the intervention group performed a regime of jumping activities for 10 minutes at the start of each physical education (PE) class, twice per week for the course of a school year. Controls undertook their usual low intensity PE warm-up. Sex-specific improvements in bone accrual were observed in jumpers, but not controls. Specifically, boys increased whole body bone mineral content (BMC) and calcaneal broadband ultrasound attenuation (BUA), and girls improved BMC at the femoral neck and spine [8]. Favourable changes in body composition were also observed [9].

Longitudinal observational data exists to support a relationship between childhood physical activity and bone mass in later life [10], [11]; however, very long term re-examination of experimental cohorts to examine maintenance of skeletal benefits into old age are required if causal inferences are to be made. As trials involving such long term monitoring from childhood into old age have limited feasibility, medium-term re-examination is the only practical alternative. A modicum of maintenance data is available for controlled exercise interventions for pre-pubertal children [12], [13], [14]. To our knowledge, the only peripubertal cohort to have been re-examined for maintenance effects of an exercise intervention involved a post-intervention period of one year and examined girls only [15].

The aim of the current study was to determine if benefits observed from the early high school POWER PE jumping intervention were maintained at school-leaving age; that is, three years after cessation of the intervention. We hypothesized that the intervention and control groups would have experienced similar gains in bone, lean and fat mass over the three-year post-intervention period such that jumping-related benefits would be sustained in the treatment group.

## Methods

### Ethics statement

Approval to conduct the study was granted by the Griffith University Human Research Ethics Committee (#PES/09/05/HREC) and Education Queensland (Department of Education and Training, Queensland Government). Written informed consent was obtained from every subject and their parent/guardian. All research activities were in accordance with the *Declaration of Helsinki*.

### Study design

The study was designed as a three-year re-examination of male and female adolescent participants in an eight-month, in-school randomized controlled trial of jumping in place of regular physical education warm ups (i.e. POWER PE). The original findings of that trial are published elsewhere [8].

Per the original intervention, testing included biometrics, maturity assessment, physical activity, diet and body composition.

### Subjects and subject selection

Participants in the POWER PE study (n = 99) were contacted and asked to volunteer for repeat testing three years after cessation of the intervention. Volunteers were included if they were of sound general health, fully ambulatory, and had the written consent of a parent or guardian. Subjects were excluded if they had an endocrine disorder, metabolic disease, or chronic renal pathology, were taking medications known to affect the musculoskeletal system, were recovering from lower limb injury, or were affected by any condition not compatible with intense physical activity.

### Biometrics

Sitting height and standing height were measured to the nearest millimetre using the stretch stature method with a portable stadiometer (HART Sport & Leisure, Australia). Weight was measured to the nearest 0.1 kilogram using digital scales (Soehnle Co., Switzerland). Body mass index (BMI) was calculated from measures of height and weight per the accepted method (BMI = weight.height^−2^, kg m^−2^).

### Physical maturity

Maturity was categorized by Tanner staging and by calculating the number of years since the age of peak height velocity (YAPHV). Tanner stage was self-determined by each subject using standard diagrams of pubic hair growth (and breast development for females). A private area was provided for completing forms, which were then placed in sealed, coded envelopes for data entry. Age of peak height velocity was calculated using chronological age and anthropometric parameters (i.e. height, sitting height, and weight) and the algorithms developed by Mirwald and colleagues [16] from a large pediatric longitudinal trial [11].

### Bone parameters and body composition

Parameters of bone strength were measured using quantitative ultrasound and dual-energy x-ray absorptiometry (DXA). Broadband ultrasound attenuation of the non-dominant calcaneus (determined as the stance leg during kicking [17]) was determined using the QUS-2 ultrasonometer (Quidel, CA). Quality control was accomplished by performing the automated calibration with a phantom block of known BUA on each day of testing. Short-term measurement precision (CV) for repeated measures of a sub-sample (n = 20) of the study cohort was 2.8%.

Whole body (WB), lumbar spine (LS) and non-dominant femoral neck (FN) and trochanter (TR) bone mineral content (BMC) was examined using an XR-46 Quickscan Densitometer (Norland Medical Systems, Inc., USA) using host software. Whole body lean tissue mass, fat mass, and percent fat were determined from WB scans. The same investigator (BW) performed and analyzed all DXA measurements. Short-term measurement precision (CV) of a sub-sample (n = 35) was 1.3%, 1.1%, and 1.4% for FN, LS, and WB BMC respectively.

### Physical activity

Past and current (previous 12 months) physical activity were quantified with the bone-specific physical activity questionnaire (BPAQ) [18]. The BPAQ is a simple, single-page questionnaire that enables the calculation of an index of bone-relevant weight-bearing exercise history by incorporating the necessary loading characteristics of magnitude, rate and frequency for each reported activity. Responses are scored using custom-designed LabVIEW software (National Instruments, Texas, USA). Intra-class correlation coefficients for inter- and intra-tester reliability for the BPAQ are very high (0.92 and 0.97, respectively) [19]. Further details of the questionnaire are published elsewhere [18].

### Calcium intake

Daily dietary calcium consumption was estimated from a calcium-focused food questionnaire. Subjects were asked to note the frequency and quantity of consumption for a large range of calcium-containing food items. Daily calcium intake (mg/day) was calculated using the *Calcium Calculator*, an internet-based java applet provided by *CalciumInfo.com* [20] that uses a database of calcium values for common foods.

### Statistical analyses

Statistical analyses were performed using SPSS version 19.0 for Windows (IBM Corporation, Somers, NY, USA). An intention-to-treat approach on the original sample (with LVCF for missing data) was employed using repeated measures ANOVAs to determine treatment effects and change across each measurement time point (i.e. 0, 8, and 44 months). One-way ANOVA was used to observe between-group differences in subject characteristics and three-year raw change in bone, muscle and fat parameters. Two-tailed Pearson correlation analyses were employed to observe relationships between physical/lifestyle characteristics and three-year change in bone, muscle and fat parameters. T-tests were used to determine if the baseline characteristics of those tested three years post-intervention differed from the complete sample tested at baseline. Statistical significance was determined at *p*≤0.05.

## Results

### Subject characteristics

A total of 29 adolescents who participated in the original trial (11 boys, 18 girls; 18 controls and 11 intervention subjects) aged 17.3±0.4 years volunteered to attend three-year retesting. Subjects were 4.2±1.0 years past APHV with 11 (38%) in Tanner stage IV and 18 (62%) in Tanner stage V. All girls were post-menarcheal with an average age of menarche of 12.8±0.7. There were no significant differences in baseline anthropometrics, bone measures, or lifestyle characteristics between the original cohort and the 29 participants tested at 44 months. Three years after the intervention, there were no significant between-group differences in subject characteristics (Table 1).

**Table 1:** Subject characteristics (mean ± SD) for controls and intervention subjects three years post-intervention (n = 29).

Characteristic	Controls	Intervention	p-value
	(n = 18)	(n = 11)	
Age (years)	17.2±0.5	17.4±0.2	0.44
Years from APHV	4.3±1.0	4.1±1.0	0.42
Weight (kg)	64.3±16.0	61.4±15.7	0.64
Height (m)	1.68±0.06	1.70±0.09	0.67
BMI (kg/m^2^)	22.5±4.4	21.1±3.7	0.65
BPAQ score	19.2±13.0	22.2±26.8	0.69
Calcium intake (mg/day)	805±319	858±281	0.65

Key: APHV, Age of peak height velocity; BMI, body mass index; BPAQ, bone-specific physical activity questionnaire.

### Bone measures

**Figure 1:**
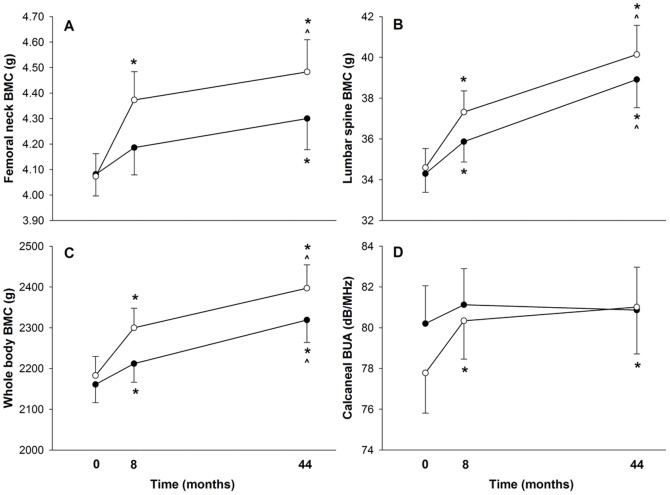
Baseline, 8-month, and 44-month femoral neck BMC (A), lumbar spine BMC (B), whole body BMC (C), and calcaneal BUA (D) for intervention (open circles) and control subjects (closed circles). Key: BMC, bone mineral content; BUA, broadband ultrasound attenuation. * represents significant difference with baseline measure. ∧ represents significant difference with 8-month measure.

Subjects in the jumping group exhibited a significant 7.4% increase in femoral neck (FN) BMC after the 8 month intervention (p = 0.001), while controls did not (+2.6%, NS) (Table 2). Three years after cessation of the intervention, both groups had increased FN BMC a further 2.5% (p≤0.05). Despite an overall improvement in intervention group FN BMC of 10.1% from baseline (p = 0.001), versus only 5.1% increase in controls (p = 0.021), a three-year between-group main effect did not reach significance (Figure 1A). The 4.8% (8 month) and 5.0% (44 month) greater increases in intervention group FN BMC than control however, were greater than the least significant change (LSC) statistic (3.6%), based on our FN measurement precision of 1.3%, indicating that the notable differences between groups were not due to measurement error.

**Table 2:** Bone and body composition values at baseline, 8 months, and 44 months (mean ± SD) for controls and intervention subjects based on intention-to-treat analysis (n = 99).

Parameter	Controls	Intervention	p-value
	(n = 52)	(n = 47)	
FN BMC at baseline (g)	4.08±0.49	4.07±0.72	0.94
FN BMC at 8 months (g)	4.19±0.52	4.37±0.96*	0.22
FN BMC at 44 months (g)	4.30±0.77*	4.48±0.96*	0.29
LS BMC at baseline (g)	34.3±5.7	34.6±7.2	0.82
LS BMC at 8 months (g)	35.9±6.5*	37.3±7.7*	0.32
LS BMC at 44 months (g)	38.9±10.4*	40.1±9.1*	0.54
WB BMC at baseline (g)	2161±253	2183±373	0.73
WB BMC at 8 months (g)	2212±254*	2300±387*	0.19
WB BMC at 44 months (g)	2319±385*	2397±393*	0.33
Calcaneal BUA at baseline (dB/MHz)	80.2±15.3	77.8±10.8	0.38
Calcaneal BUA at 8 months (dB/MHz)	81.1±14.6	80.3±10.3*	0.76
Calcaneal BUA at 44 months (dB/MHz)	80.9±18.6	81.0±11.2*	0.96
Lean mass at baseline (g)	31993±4220	34699±7111	0.02
Lean mass at 8 months (g)	32974±5148*	36993±7591*	0.01
Lean mass at 44 months (g)	36077±8869*	39171±8362*	0.08
Body mass index at baseline (kg/m^2^)	20.0±3.5	20.0±3.5	0.98
Body mass index at 8 months (kg/m^2^)	20.4±3.3*	20.5±3.3*	0.90
Body mass index at 44 months (kg/m^2^)	21.1±4.0*	20.7±3.3	0.65
Fat mass at baseline (g)	17245±4984	17763±7163	0.68
Fat mass at 8 months (g)	17220±5337	17332±7234	0.93
Fat mass at 44 months (g)	18410±6137*	17919±7549	0.72
Percent fat mass at baseline	25.5±5.3	25.0±7.3	0.74
Percent fat mass at 8 months (g)	24.9±5.9	23.5±7.7*	0.32
Percent fat mass at 44 months (g)	25.0±5.7	23.3±8.0*	0.21

Key: BUA, broadband ultrasound attenuation; FN BMC, femoral neck bone mineral content; LS BMC, lumbar spine bone mineral content; WB BMC, whole body bone mineral content.

* represents significant difference with baseline measure.

Both groups experienced significant gains in LS BMC during the intervention year (Int = +7.9%, p = 0.001; Ctrl = +4.6%, p = 0.001). While no significant between-group main effect could be detected, the 3.3% greater increase in intervention group LS BMC than control exceeded the LSC statistic (3.1%) based on our LS measurement precision of 1.1%. During the three-year follow-up period, both groups increased LS BMC a similar amount (Int = +7.9%, p = 0.004; Ctrl = +8.4%, p = 0.001) such that final values for both groups were significantly greater than baseline (Int = +15.8%, p = 0.001; Ctrl = +13.5%, p = 0.001). Thus the trend for greater LS BMC gain in intervention group than controls at 8 months appeared to be sustained at 44 months; however those differences did not reach significance (Figure 1B); nor did the between-group difference in 44-month change (2.8%) exceed the least significant change statistic.

WB BMC at 44 months was significantly greater than baseline in both groups (Int = +9.6%, p = 0.001; Ctrl = +7.2%, p = 0.001). The trend for greater gain in intervention group WB BMC than control at 8 months (Int = +5.4%, p = 0.001; Ctrl = +2.4%, p = 0.001) was sustained at 44 months owing to similar WB BMC gains in both groups over the post-intervention period (Int = +4.2%, p = 0.005; Ctrl = +4.8%, p = 0.001). The between-group difference, however, did not reach significance at either time point (Figure 1C) or exceed the least significant change statistic (3.9%) based on our WB measurement precision of 1.4%.

The intervention group increased calcaneal BUA by 3.3% at 8 months (p = 0.005), compared with no change in controls (+1.2%, NS). As neither group increased calcaneal BUA in the post-intervention period (Figure 1D), intervention group calcaneal BUA was ultimately 4.2% greater than baseline at 44 months (p = 0.05), but unchanged in controls (+0.8%, NS).

### Lean mass

**Figure 2:**
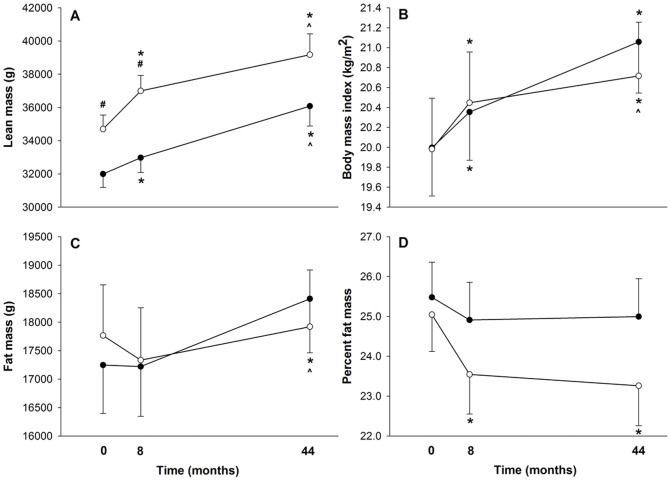
Baseline, 8-month, and 44-month lean mass (A), body mass index (B), fat mass (C), and percent fat mass (D) for intervention (open circles) and control subjects (closed circles). * represents significant difference with baseline measure. ∧ represents significant difference with 8-month measure. # represents significant between-group difference.

Final lean mass was significantly greater than baseline in both groups (Int = +12.9%, p = 0.001; Ctrl = +12.8%, p = 0.001). A trend for greater lean mass gain existed for the treatment group immediately following the intervention (Int = +6.6%, p = 0.001; Ctrl = +3.0%, p = 0.001), but the reverse trend was apparent at the three-year post-intervention period (Int = +5.9%, p = 0.008; Ctrl = +9.4%, p = 0.001), and no differences were significant. Moderate positive relationships were observed between three-year change in bone-free lean mass and three-year change in FN BMC (r = 0.56, p = 0.01) and LS BMC (r = 0.64, p = 0.01) (Figure 2A).

### BMI and Fat mass

Both groups increased body mass index (BMI) during the intervention period (Int = +2.3%, p = 0.001; Ctrl = +1.8%, p = 0.006). During the three years following the intervention, control group BMI continued to increase significantly (+3.5%, p = 0.001), but intervention group BMI did not (−1.3%, p = 0.14). BMI for both groups was significantly greater at 44 months than at baseline (Int = +3.7%, p = 0.001; Ctrl = +5.3%, p = 0.001) with no significant differences between groups (Figure 2B).

There was no significant change in absolute fat mass for either group during the intervention period (Int = −2.4%; Ctrl = −0.1%); however intervention subjects decreased percent fat significantly (−6.0%, p = 0.001), versus no change in controls (−2.3%, p = 0.051). Controls significantly increased fat mass in the three-year post-intervention period (+6.9%, p = 0.002), but the intervention group did not (+3.4%, NS). As a consequence, fat mass at 44 months was greater than baseline for controls only (+6.8%, p = 0.01) (Figure 2C). Neither group experienced any significant change in percent fat during the three-year post-intervention period (Int = −1.2%, p = 0.43; Ctrl = +0.3%, p = 0.81) (Figure 2D).

## Discussion

It is commonly espoused that osteogenic exercise in youth is a logical strategy to prevent osteoporotic fracture in old age. In fact, only very few studies have examined whether exercise benefits to the pediatric skeleton are sustained to any degree after cessation of an exercise intervention. Our goal, therefore, was to determine if the positive bone, muscle and fat changes from an eight-month, in-school, adolescent, jumping intervention were sustained three-years after cessation of the activity. Consequently, although the original skeletal improvements at the hip, spine, whole body, and calcaneus in the jumping group were sustained, group differences did not reach significance at 44 months. Of note, a trend for greater accumulation of fat was observed in controls at 44 months.

Previous pediatric exercise interventions have predominantly targeted prepubertal children, to the extent that very little adolescent data has been reported. Given the reported relationship of physical activity and bone to maturity [21], examination of exercise effects in older children was warranted. The POWER PE trial [8] was designed to specifically target peripubertal adolescents.

Some evidence suggests that the osteogenic benefits of exercise in childhood can be sustained at the femoral neck [13], [14], lumbar spine [14], and tibia [12]. The examination of only short duration (i.e. one year) maintenance [13], prepubertal cohorts [12], [13], [14], and single sex participants [15], however, limit the generalizability of those observations. A single trial has examined maintenance of skeletal changes in an adolescent cohort of girls. Those data indicated that lumbar spine bone gains following a nine-month jumping intervention were maintained for 12 months [15]. Our data provides additional evidence that 1) the skeletal benefits of brief jumping bouts during adolescence may endure for at least three years, 2) trends are also evident at the femoral neck and whole body, and 3) the effect is apparent when inclusive of boys and girls. While a group-by-time main effect could not be detected (likely due to low subject numbers), we found that the greater absolute values of bone mass attained by jumpers than controls immediately post intervention were sustained by a paralleling of bone growth trajectories during the following three years. Notably, our results show that the apparent maintenance effect endures from the time of peak height velocity until a time nearing the achievement of peak height.

Despite the known inadequacies of BMI as an index of body composition or fatness, we chose to include it as an outcome measure for the purpose of comparison with others' work. While waist circumference is a better predictor of obesity-related disease than BMI or weight [22], BMI has nevertheless been associated with coronary heart disease [23], metabolic syndrome [24], and progression of osteoarthritis of the knee [25]. Although there was no significant between-group difference in BMI at 44 months, a trend for lower BMI in jumpers was observed, reflecting perhaps a divergent pattern of lean and fat tissue development between groups following the intervention.

That positive skeletal changes were observed in the original POWER PE trial suggests the window of opportunity for eliciting bone benefits during childhood remains open well into puberty. Given that peak bone mass is typically achieved in the third decade life, it is possible that the temporal opportunity to enhance skeletal strength may be greater than is generally accepted. In fact, we have previously shown that calcaneal BUA of young adult men maintains an upward trajectory through 25 years of age [26]. Utilising exercise then to optimise peak bone mass remains a potentially effective strategy into young adulthood. Although some data exists, it has not been definitively established if late uptake of exercise in young adulthood can be notably osteogenic, nor whether any such late onset bone gains would be maintained over the long term.

The primary study limitation to be acknowledged is the considerable subject drop-out between 8-month and final 44-month measures. As is typical during long-term randomised controlled trials, participant relocations and loss of interest accounted for the majority of loss. There were no difference in baseline characteristics between subjects who dropped out and those who returned for three-year retesting. The issue was managed statistically with the use of intention to treat analysis and the imputation of missing values. Unlike our original study, our remaining sample size was not sufficient to explore sex-specific outcomes and thus, our investigation was based on a group analysis only.

In conclusion, while between-group differences were not significant at 44 months, our findings suggest that osteogenic benefits of an 8-month in-school jumping intervention for adolescents can be sustained for at least three years (into young adulthood) and that positive trends in fat tissue mass may also occur. Furthermore, those trends can be achieved from a very simple, twice-weekly 10 minute intervention amenable to high school implementation with no disruption to existing physical education activities.
